# Water quality attribution and simulation of non-point source pollution load flux in the Hulan River basin

**DOI:** 10.1038/s41598-020-59980-7

**Published:** 2020-02-20

**Authors:** Yan Liu, Hongyan Li, Geng Cui, Yuqing Cao

**Affiliations:** 1 Key Laboratory of Groundwater Resources and Environment (Jilin University), Ministry of Education, Changchun, 130021 China; 2 Jilin Provincial Key Laboratory of Water Resources and Environment, Jilin University, Changchun, 130021 China; 3 Northeast Institute of Geography and Agroecology, Chinese Academy of Sciences, Changchun, 130102 China

**Keywords:** Geochemistry, Hydrology

## Abstract

Surface water is the main source of irrigation and drinking water for rural communities by the Hulan River basin, an important grain-producing region in northeastern China. Understanding the spatial and temporal distribution of water quality and its driving forces is critical for sustainable development and the protection of water resources in the basin. Following sample collection and testing, the spatial distribution and driving forces of water quality were investigated using cluster analysis, hydrochemical feature partitioning, and Gibbs diagrams. The results demonstrated that the surface waters of the Hulan River Basin tend to be medium–weakly alkaline with a low degree of mineralization and water-rock interaction. Changes in topography and land use, confluence, application of pesticides and fertilizers, and the development of tourism were found to be important driving forces affecting the water quality of the basin. Non-point source pollution load fluxes of nitrogen (N) and phosphorus (P) were simulated using the Soil Water and Assessment Tool. The simulation demonstrated that the non-point source pollution loading is low upstream and increases downstream. The distributions of N and P loading varied throughout the basin. The findings of this study provide information regarding the spatial distribution of water quality in the region and present a scientific basis for future pollution control.

## Introduction

Rivers are an important component of the global water cycle, connecting the two major ecosystems of land and sea and providing a critical link in the biogeochemical cycle. The spatial distribution of water quality is indicative of the environment in which a river flows. River chemical composition is influenced by natural factors such as climate, lithology, soil, vegetation, and anthropogenic activities. Hence, studies on river water chemical characteristics can provide important information on geochemical behavior, rock weathering, and human activities in a basin^1–3^.

Over the last 50 years, scholars have studied the water chemistry of major rivers in all continents and have explored the main forces affecting water chemistry in river basins^4,5^. For instance, the main driving forces affecting river water chemistry include land use and land cover changes^6–10^, rainfall intensity, pollution build-up levels, wastewater discharges, and anthropogenic influences^11–13^.

Pollution inputs to surface water result in the evolution and deterioration of river water quality. Pollutants can be classified as point source and non-point source. Point source pollution is relatively easy to adjust and control because it is easy to monitor their concentration and flux^14^. Conversely, non-point sources often come from extensive areas of land and can be transported overland, underground, or even through the atmosphere to receiving water bodies^15^, making them difficult to measure and control. Non-point source pollution, mainly nitrogen (N) and phosphorus (P), has led to excessive nutrient inputs and surface water quality decline.

In China, human activities have resulted in widespread water quality deterioration, directly impacting the overall ecological environment and socioeconomic development. The contribution of non-point source N and P pollution to total water pollution in China has been found to be as high as 81% and 93%, respectively^16^.

Agriculture and urban life are the main sources of N and P in aquatic ecosystems. Atmospheric deposition is also an important source of N. Non-point source inputs of these pollutants are difficult to measure and adjust because these elements come from various human activities that are distributed over a large area. Temporal changes due to the influence of weather also contribute to this difficulty. In aquatic ecosystems, N and P can cause the proliferation of toxic algae, anoxia, fish deaths, biodiversity loss, and the loss of aquatic plant beds and coral reefs. Eutrophication severely impacts aquatic ecosystems and threatens water use for drinking, industry, agriculture, and recreation^17,18^.

In recent decades, the basin hydrological model has developed rapidly, and models considering hydrological and sediment transport processes in complex basins have emerged. Among them, the Soil and Water Assessment Tool (SWAT) model^19^ is well known. The SWAT model is widely used in the assessment of hydrological sediment and pollutant migration processes at the basin scale. It employs a number of factors, including meteorological data, underlying surfaces, and human management measures, to effectively simulate surface runoff, groundwater, sediment transport, and non-point source pollution^20–22^.

The SWAT model can be used to simulate changes in hydrological sediment and non-point source pollution in a variety of vegetation cover and land use types including forest cover^23^, biodiversity-rich areas^24^, and highly developed agricultural regions^25^. At the same time, the SWAT model can simulate the impact of climate change on the water environment processes of the basin. More importantly, the SWAT model can effectively simulate the melting of snow and the process of glacial snowmelt^26^. The model has been successfully applied to rainfall^27,28^ and snowmelt events^29^.

For mid to high latitudes, minimum temperatures are generally below 0 °C from October–April, during which the water and soil in the basin have freeze-thaw cycles. Therefore, using the SWAT model to simulate hydrological, soil erosion, and pollutant migration processes in mid to high latitude freeze-thaw areas can produce good results, and play an important role in the study of water and soil resources and environmental effects evaluation.

The Hulan River is a tributary of the Songhua River. The Hulan River basin is a productive agricultural area and is an important commodity grain base in the fertile Heilongjiang Province. The Hulan River is the main source of farmland irrigation in the region; hence, river water quality impacts food quality and community health. Non-point source pollution has become the main source of pollution in basin waters because of agriculture’s large outputs of N and P. This affects the region’s industrial structure and ecological environment. For this reason, our study has two objectives: (1) to analyze the spatial distribution characteristics of surface water quality and its driving factors in the basin and (2) to simulate the non-point source pollution load flux of the main pollutants (nitrogen and phosphorus) in the basin. The study of watershed chemical characteristics and simulations of non-point source pollution load flux will provide a scientific basis for the effective control of non-point source pollution, water pollution, improvement of the water environment, and for the comprehensive planning of Hulan River basin water conservation.

## Methods

### Study area

**Figure 1:**
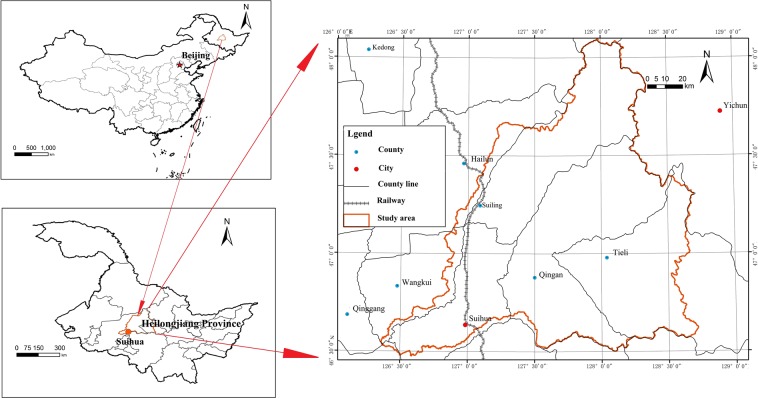
Location of the Hulan River Basin in eastern Songnen Plain in the central Heilongjiang Province, China.

The Hulan River Basin is located in the eastern Songnen Plain in the central Heilongjiang Province (Fig. 1). It is a primary tributary of the left bank of the Songhua River, located from 125°55′–128°43′ east and from 45°52′–48°03′ north. The basin is approximately 240 km from north to south, and 210 km from east to west.

**Figure 2:**
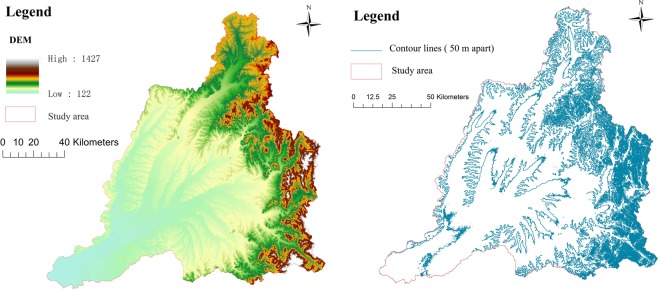
Topography and elevation contours of the Hulan River Basin.

The terrain of the Hulan River Basin is fan-shaped. The east region is mountainous, belonging to the Xiaoxing’anling Mountains, a forestry production base with dense forests. The western and central region are hilly terraces with elevations of 200–300 m and ground slopes of approximately 1/20–1/200. The southern region is low-lying, with elevations between 120–200 m and ground slopes of approximately 1/200–1/3000. The terrain slopes from the northeast to southwest, as shown in Fig. 2.

The high latitude of the Hulan River Basin means that the region has a cold temperate continental monsoon climate with an average annual temperature of 0–3 °C. Owing to mountain airflow uplift, precipitation decreases from east to west. The average annual precipitation is approximately 700 mm in the east and 500 mm in the west. The average annual basin runoff is 40.98 billion m^3^, and the average annual runoff depth is 114.8 mm.

The geological structures in the study area are formed through the processes of fault depression, depression and shrinkage. The Mesozoic is dominated by fault depression, forming a basement. The Mesozoic and Cenozoic are dominated by sedimentation and depression, forming a caprock. Inland river and lake deposits of the Mesozoic and Cenozoic Erathem with a thickness of about 8000 m were deposited in the study area. Generally, the strata of the study area are divided into Cretaceous, Paleogene, Neogene and Quaternary.

**Figure 3:**
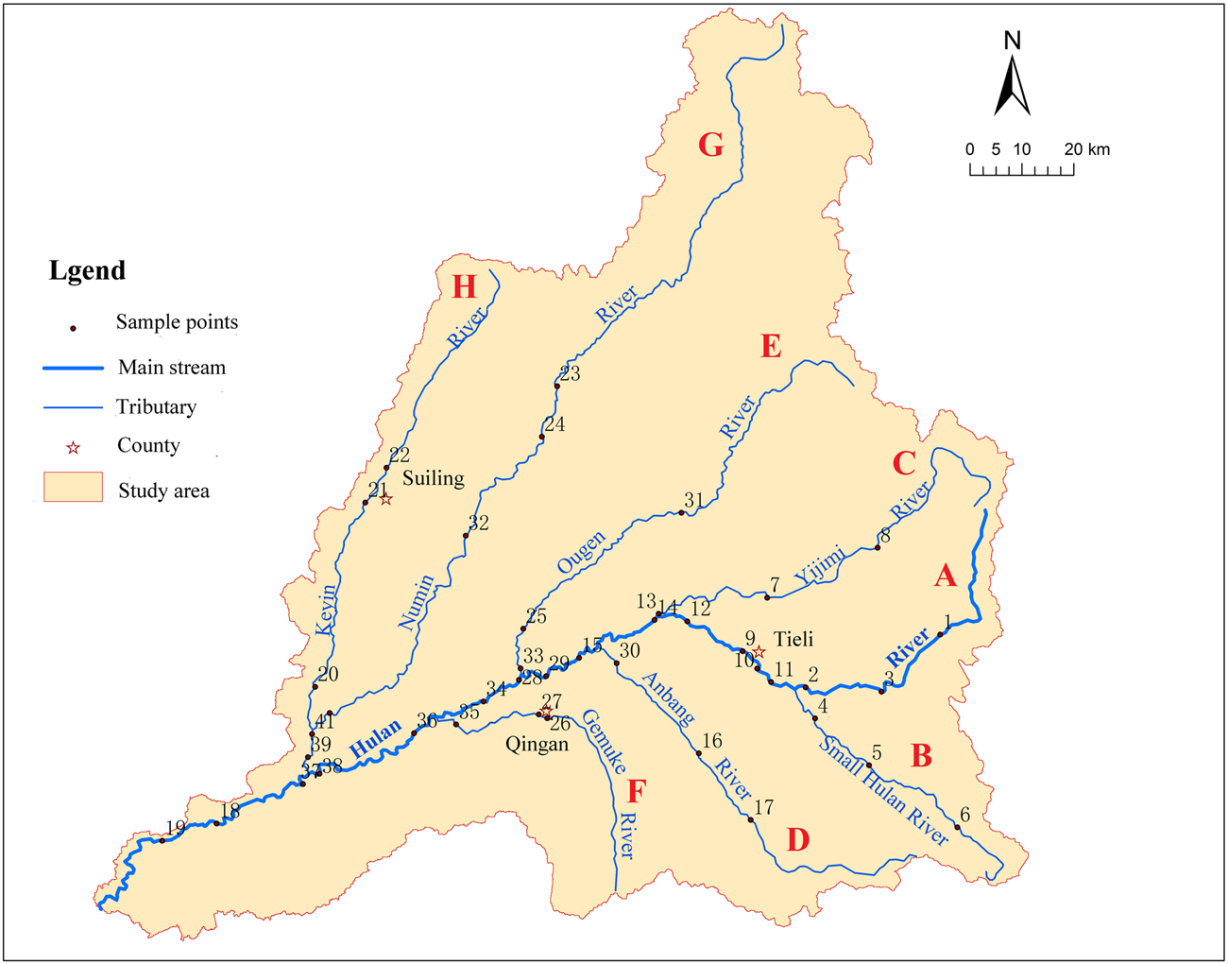
Water sampling point in main stream and tributaries of the Hulan River basin (Symbolizing each river by capital letters A to H from upper reaches to lower reaches, the same below).

The Hulan River originates from the Luchui Mountain in the northeast of Tieli City on the west side of Xiaoxing’anling with a maximum elevation of 920 m. It flows from east to west with a total length of 523 km. The south bank has tributaries, including the Xiaohulan, Anbang, and Gemuke Rivers, while the north bank includes the Yijimi, Ougen, Numin, and Keyin Rivers. The Hulan River meets with the Tongken River, which flows from the north to the south in Tongjiang, Wangkui County, then turns to the south. The river system is finally injected into the Songhua River to the southeast of Lanhe, Hulan County as shown in Fig. 3.

### Sample collection and analysis

Based on existing survey data and according to the distribution of the water system and land use in the survey area, field sampling of the Hulan River Basin was conducted during June and October 2018. Sampling points were distributed in the upper, middle, and lower reaches of the river, both upstream and downstream of confluences and cities. The spatial distribution of sampling points is shown in Fig. 3.

Water samples were collected according to the “Technical Specifications for Surface Water and Sewage Monitoring” (HJ/T91–2002). An HQ40d Hach water quality monitor was used to test water temperature, total dissolved solids (TDS), conductivity, dissolved oxygen, and redox potential. Water was stored at 0–4 °C and total nitrogen (TN), total phosphorus (TP), chemical oxygen demand (CODCr), and ionic composition were measured within eight hours. TN was determined using alkaline potassium persulfate digestion UV spectrophotometry (GB11894-89), TP was determined using ammonium molybdate spectrophotometry (GB11893-89), CODCr was determined using the dichromate method (GB11914-89).

### Data analysis

The statistical analysis of water quality indicators was conducted using SPSS. A cluster analysis and principal component dimensionality reduction were used to determine the spatial difference and similarity of water quality. Water chemistry type was determined according to the Shukalev classification^30^, and pollutant sources were analyzed using the end element map^1^ and Gibbs diagrams^4^.

### SWAT model

The SWAT model is a process-based continuous distributed watershed hydrological model developed by the US Department of Agriculture Agricultural Research Center (USDA-ARS) on the basis of the Simulator for Water Resources in Rural Basins model during the 1990s^22^. The SWAT model is used for basin wide simulations of surface source pollution; water resources assessment and management; soil and water conservation; prediction of the influence of climate change; and land management measures on hydrology, sediment and nutrient production, and migration in complex watersheds. The SWAT model is divided into four modules; hydrological, soil erosion and sediment transport, nutrient transport, and plant growth and management. The nutrient transport module of the SWAT model simulates the migration and transformation of N and P nutrients. The migration and transformation of N, particularly NO_3_ contained in runoff, lateral flow, and infiltration, are calculated by the volume of water and the average degree of aggregation. Effects of filtration are considered for underground infiltration and lateral runoff. Nitrogen can be divided into dissolved N and adsorbed N, where dissolved nitrogen is mainly nitrate N. Before calculating the total amount of nitrate N, it is necessary to calculate the concentration of nitrate N in mobile water, and then multiply the concentration by the amount of water to obtain the total amount of nitrate N. The calculation of free water nitrate N concentration is as follows:

1 $${{\rm{\rho }}}_{mobile}=\frac{{\rho }_{ly}\cdot \exp [\frac{-{w}_{{\rm{moble}}}}{(1-{\theta }_{e})SA{T}_{ly}}]}{{{\rm{w}}}_{{\rm{mobile}}}}$$

where p_mobile_ is the concentration of nitrate N in free water (kg/mm), p_ly_ is the amount of nitrate N in the soil (kg/hm^2^), W_mobile_ is the amount of free water in the soil (mm), θ_e_ is porosity, and SAT is the soil saturated water content.

Adsorbed N is mainly organic N and is determined using the model developed by McElroy *et al*. and modified by Williams and Hann^31^. The expression is:

2 $${{\rm{\rho }}}_{{{\rm{orgN}}}_{{\rm{surf}}}}=0.001\times {{\rm{\rho }}}_{{\rm{orgN}}}\cdot \frac{m}{{A}_{hru}}\cdot {{\rm{\varepsilon }}}_{N}$$

where ρ_orgNsurf_ is the amount of organic N loss (kg/hm^2^), ρ_orgN_ is the concentration of organic N in the soil surface layer to a depth of 10 mm (kg/t), m is the amount of soil loss (t), A_hru_ is the area of the hydrological response unit (hm^2^), and ε_N_ is the nitrogen enrichment coefficient (dimensionless).

Phosphorus is also divided into dissolved P and adsorbed P. The migration of dissolved P in the soil is mainly achieved by diffusion. Since dissolved P is not very active, the surface layer of P in dissolved form is rarely removed from surface runoff. Dissolved P transported by surface runoff is calculated by:

3 $${P}_{{\rm{surf}}}=\frac{{P}_{solution,surf}\cdot {Q}_{surf}}{{\rho }_{b}\cdot {h}_{surf}\cdot {k}_{d,surf}}$$

where P_surf_ is dissolved P lost through surface runoff (kg/hm^2^), P_solution,surf_ is dissolved P in soil (kg/hm^2^), ρ_b_ is soil bulk density (mg/m^3^), A_hru_ is surface soil depth (mm), and k_d,surf_ is the soil P partition coefficient (dimensionless).

Adsorbed P is mainly divided into organic P and mineral P, which are usually adsorbed on soil particles and migrate with runoff. The calculation expression is:

4 $${m}_{{P}_{surf}}=0.001\times {\rho }_{P}\cdot \frac{m}{{A}_{hru}}{\varepsilon }_{P}$$

where m_Psurf_ is the amount of organic P loss (kg/hm^2^), ρ_P_ is the concentration of organic P in the surface soil (kg/t), m is the amount of soil loss (t), A_hru_ is the area of the hydrological response unit (hm^2^), and ε_P_ is the P enrichment factor (dimensionless).

## Results

### Water quality characteristics

**Figure 4:**
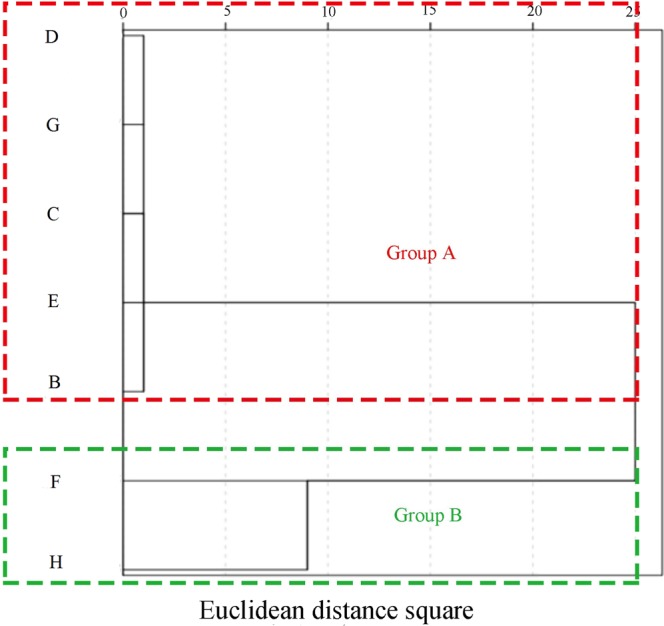
Water quality spatial clustering analysis results for tributaries of the Hulan River.

The cluster analysis and principal component dimensionality reduction analysis were used to classify rivers in Hulan basin based on the spatial distribution of pollutants. Average values of water quality indicators for the seven tributaries flowing into the main stream of the Hulan River Basin were clustered using squared Euclidean distance as the clustering index and results are shown in Fig. 4. Tributaries can be divided into two groups—A: the Anbang, Numin, Yijimi, Ougen, and Small Hulan Rivers, and B: the Keyin and Gemuke Rivers.

**Figure 5:**
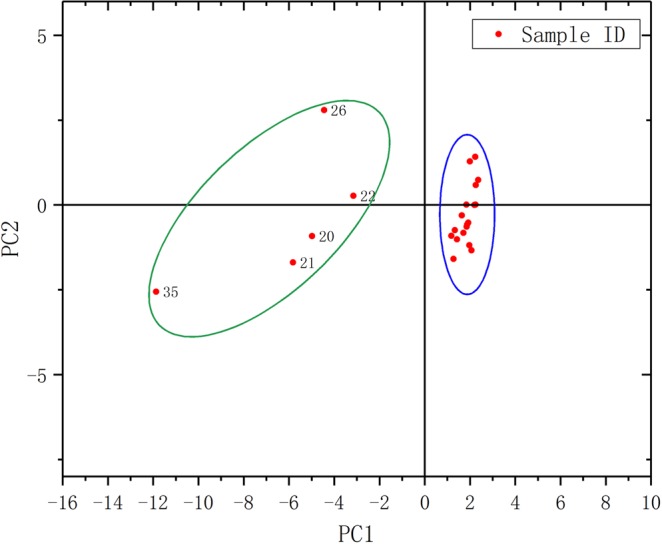
Scatter plot showing the principal component analysis of river water quality sample clusters.

Figure 5 shows that the water quality of the sampling points can be divided into two groups, with green areas belonging to the sampling points of the Keyin and Gemuke Rivers. Sampling points 39 and 41 downstream of the Keyin River are not in this grouping due to the influx of other tributaries, which impact water quality. The water quality samples in the blue region are relatively similar and represent the sampling points of the remaining tributaries, corresponding well to the results of the cluster analysis.

Based on the spatial distribution of water quality, we divide the basin into Group A (Ampang, Numin, Yijimi, Ougen, and Small Hulan Rivers), Group B (Keyin and Gemuke Rivers), and the main stream of the Hulan River.

**Figure 6:**
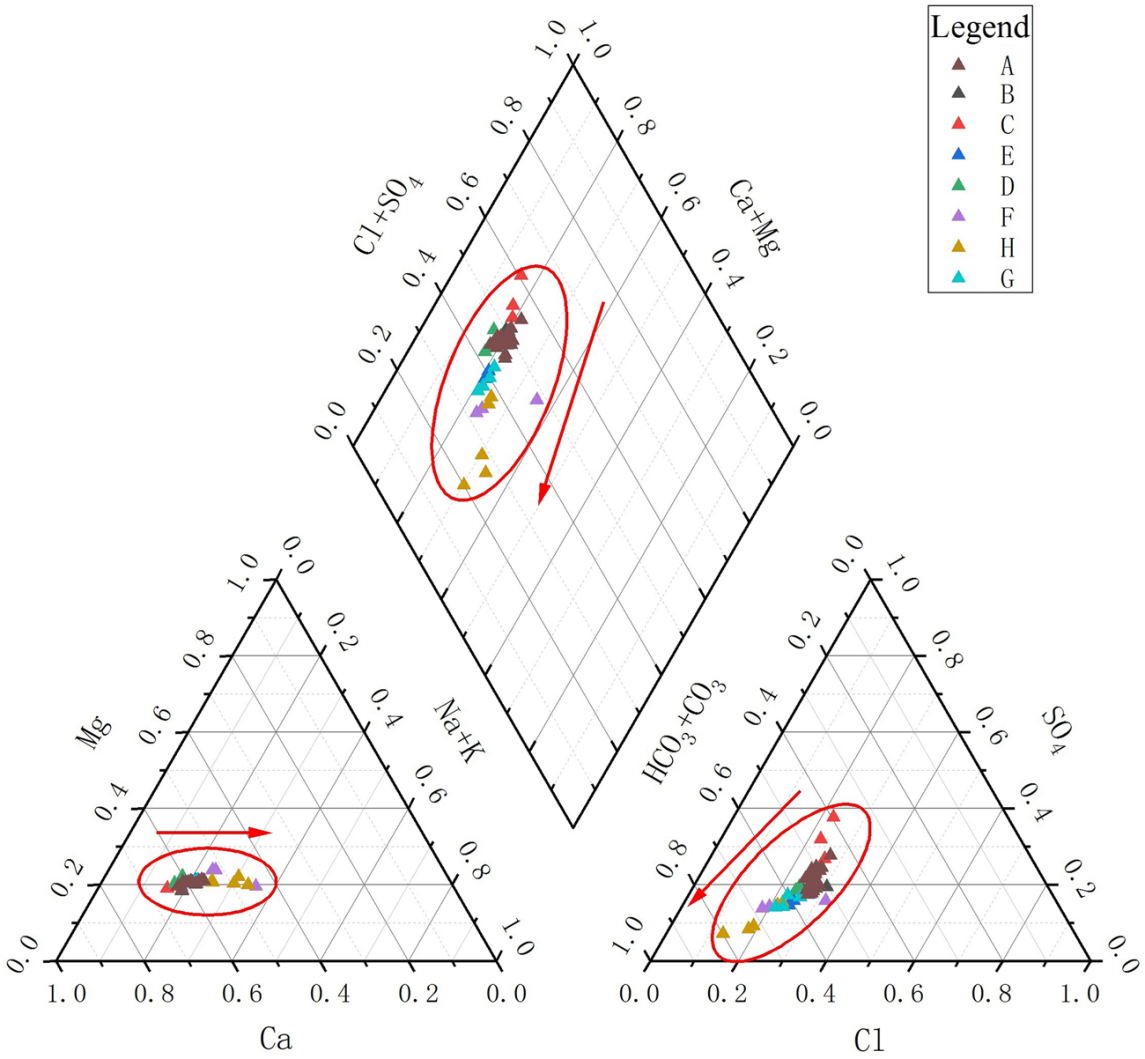
Piper diagram of surface waters in the Hulan River basin showing water chemistry types.

**Figure 7:**
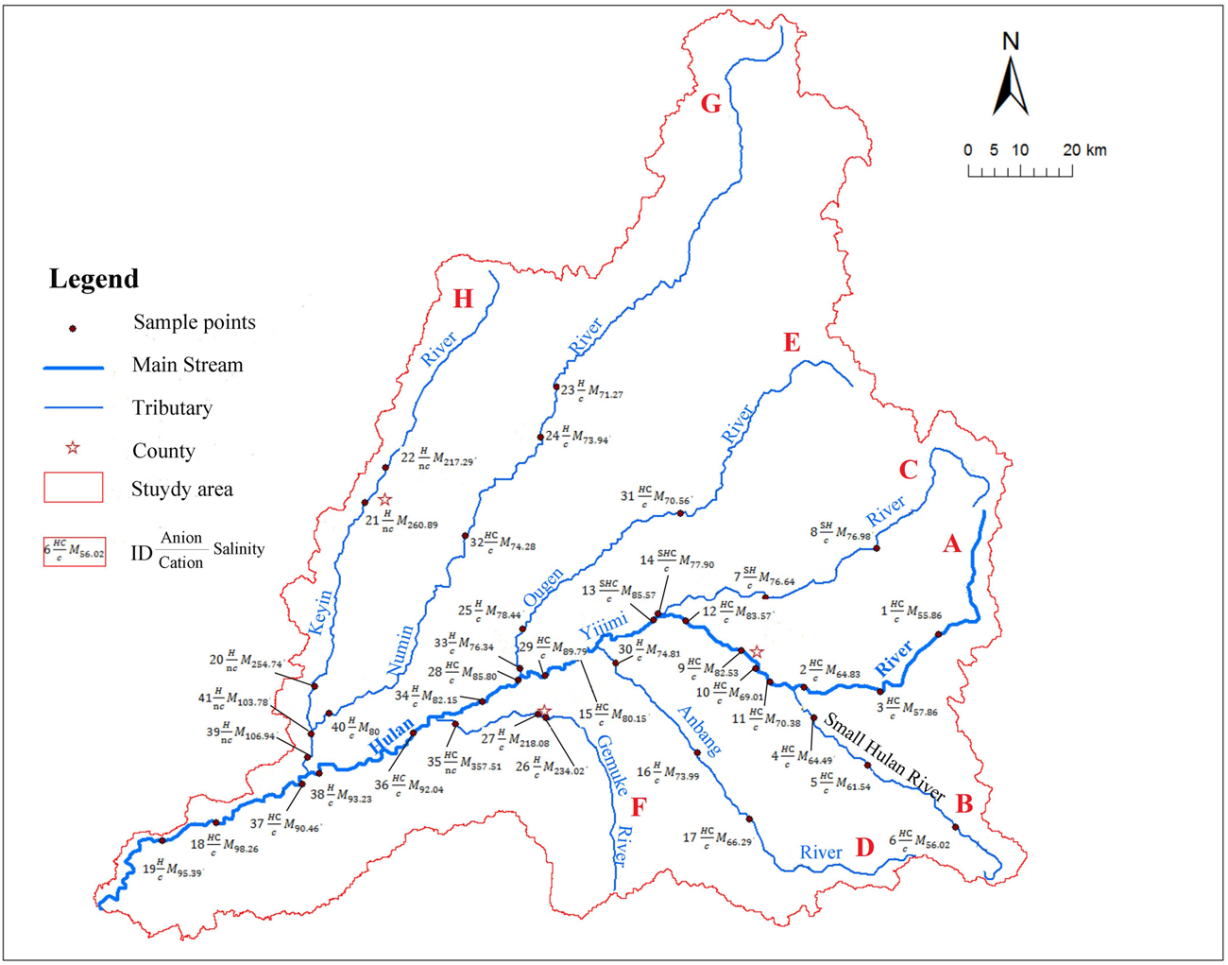
Spatial distribution of water chemistry type.

Water chemistry determined using the Shukalev classification method are shown in the Piper three-line diagram (Fig. 6) and the water chemistry type zoning diagram (Fig. 7).

It can be seen from the piper diagram that the water chemistry type of the Hulan River main stream is mainly HCO3-Ca, while that of the tributary from upstream to downstream within the basin changes from HCO3•SO4-Ca to HCO3-Ca•Mg.

The TDS of Group A is 50~80 mg/L and water chemistry type is predominantly HCO_3_•Cl-Ca(HCO_3_-Ca); however, the water type of the Yijimi River is SO_4_•HCO_3_-Ca. The TDS of Group B is 91~298 mg/L, and the water chemistry types are HCO_3_-Ca and HCO_3_•Na-Ca. The average TDS value of the Hulan River main stream is 55~95 mg/L, and water chemical types are HCO_3_•Cl-Ca and HCO_3_-Ca. Downstream of the Yijimi River injection, the water type is SO_4_•HCO_3_•Cl-Ca. The TDS of surface water in the basin is generally low, increasing gradually downstream except for in the Keyin River following the injection of the Numin River. The TDS of Group B is much higher as compared to Group A, while the Hulan River main stream has a TDS between these two groups. The ion types of the rivers in the basin are gradually enriched downstream.

The average concentration of the major ions in basin surface waters is shown in Table 1. It is apparent that ion concentrations in Group A are lower as compared to Group B, while the ion concentrations of the main stream are between these two groups. Ion concentrations generally increase downstream. Anion concentrations in Groups A and B are HCO_3_^−^ > Cl^−^ > SO_4_^2−^ and HCO_3_^−^»SO_4_^2−^ > Cl^−^, respectively, while all groups have cation concentrations Ca^2+^ > K^+^ + Na^+^ > Mg^2^+. Overall, HCO^3−^ and Ca^2+^ are the dominant components.

**Table 1:** Average ion content of each river groups A and B and the mainstream.

Region	Location	Average ion content (mg/L)					
		Na^+^ + K+	Ca^2+^	Mg^2+^	HCO_3_^−^	SO_4_^2−^	Cl^−^
Group A	Upstream	5.16	14.42	2.67	31.40	12.18	10.01
	Midstream	13.19	25.91	6.44	79.84	16.74	15.64
	Downstream	17.34	25.03	5.93	67.34	17.78	21.41
Group B	Upstream	15.64	25.06	5.90	97.99	10.41	12.87
	Midstream	24.79	31.84	7.91	122.68	14.21	20.31
	Downstream	17.86	27.08	6.82	96.55	14.46	17.43
Mainstream	Upstream	6.26	14.85	3.00	35.36	10.79	10.77
	Midstream	8.49	18.41	3.96	42.99	15.73	12.69
	Downstream	10.11	19.66	4.44	49.78	16.32	14.06

Comprehensive environmental indicators characterize the overall salinity of the water body, including total hardness and conductivity. Oxidation reduction potential (Eh), pH, dissolved oxygen (DO), and biochemical oxygen demand (COD) are shown in Table 2. Total hardness and conductivity generally show an increasing trend downstream and Group B values are much higher as compared to Group A and the mainstream. The pH values of surface waters are between 7.51 and 8.09 (medium-weak alkaline water) and Group B pH is slightly higher as compared to the other two regions. The pH of the main stream increases gradually downstream, while Group A and B pH decreases gradually. The Eh of the surface water in the basin is 200 ± 10 mv. According to the “Environmental Quality Standard for Surface Water” (GB3838-2002), most of the dissolved oxygen (DO) levels meet Class I and II water quality standards, and very few sites are Class III (the middle reaches of the Keyin and Hulan Rivers). The COD of most surface waters is in the IV and V standard range, while the middle reaches of the Gemuke and Hulan Rivers are nearly twice as high as the Class III standard.

**Table 2:** Water quality environmental indicators.

Region	Location	Total hardness (mg/L, calculated as CaCO_3_)	Electrical conductance (μs/cm)	Eh (mV)	DO (mg/L)	CODcr (mg/L)	pH
Group A	Upstream	49.69	114.67	209.83	7.21	23.44	7.76
	Midstream	56.42	129.00	200.00	7.65	23.00	7.63
	Downstream	57.33	136.00	195.60	7.93	23.80	7.60
Group B	Upstream	155.79	365.00	183.00	7.45	30.29	8.09
	Midstream	171.16	379.33	171.33	7.70	34.52	7.89
	Downstream	137.62	294.75	197.75	7.39	22.18	7.57
Mainstream	Upstream	50.01	114.14	170.25	6.65	23.96	7.51
	Midstream	63.00	150.17	196.00	7.44	21.43	7.64
	Downstream	68.20	163.67	203.67	6.93	22.26	7.66

**Figure 8:**
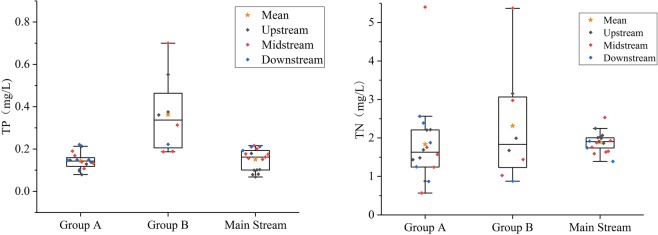
Spatial characteristics of total nitrogen and phosphorus in the Hulan River basin.

Based on the China’s drinking water hygiene standards (GB 5749-2006), according to the Class III water standard, the most polluting components in the basin are total N and P. Figure 8 shows that the water quality of total N is generally Class IV and V, while some samples are inferior to Class V. The total N content in Group B is higher as compared to Group A and the Hulan mainstream. The total P content in Group B is higher as compared to Group A and the Hulan mainstream.

### Driving forces of water quality

**Figure 9:**
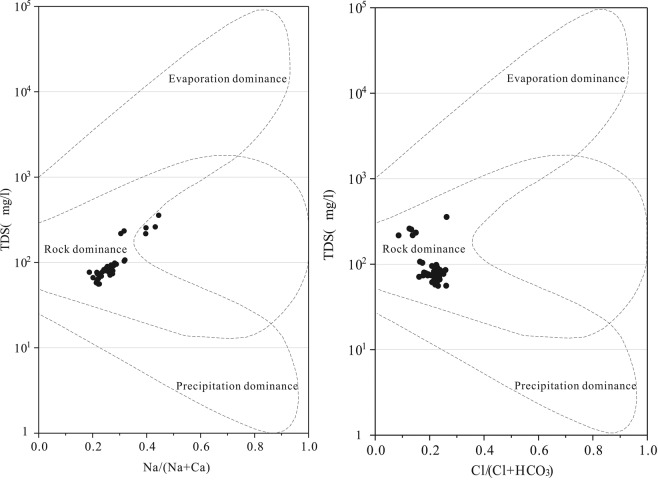
Gibbs diagrams of surface water chemical origins.

Figure 9 shows Gibbs diagrams of the basin. It is apparent that samples generally fall in the rock weathering control area, indicating that the water chemistry of the basin is mainly controlled by rock weathering^4,32,33^.

**Figure 10:**
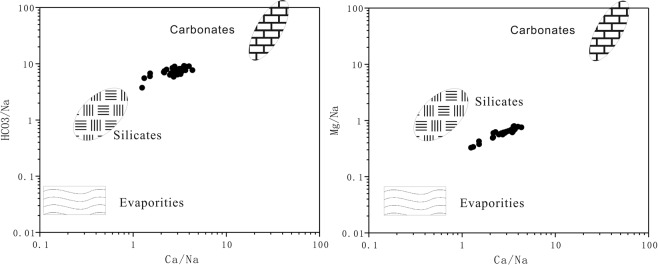
End element plots indicating the origins of ions in surface waters.

Figure 10 shows that ions are mainly composed of silicate mineral weathering products, followed by carbonate mineral weathering products, corresponding well with the geological features of the region.

**Figure 11:**
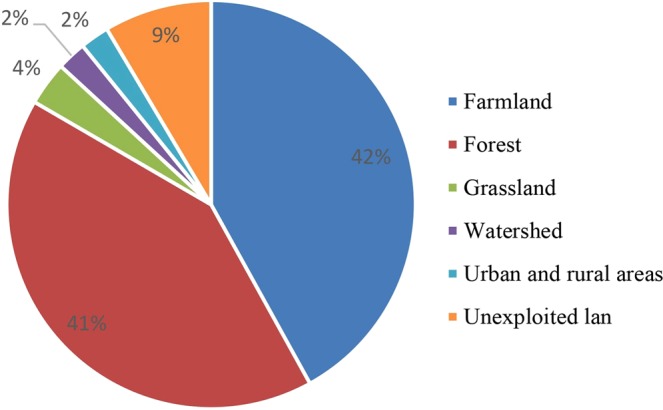
Pie chart showing the proportion of different land uses.

**Figure 12:**
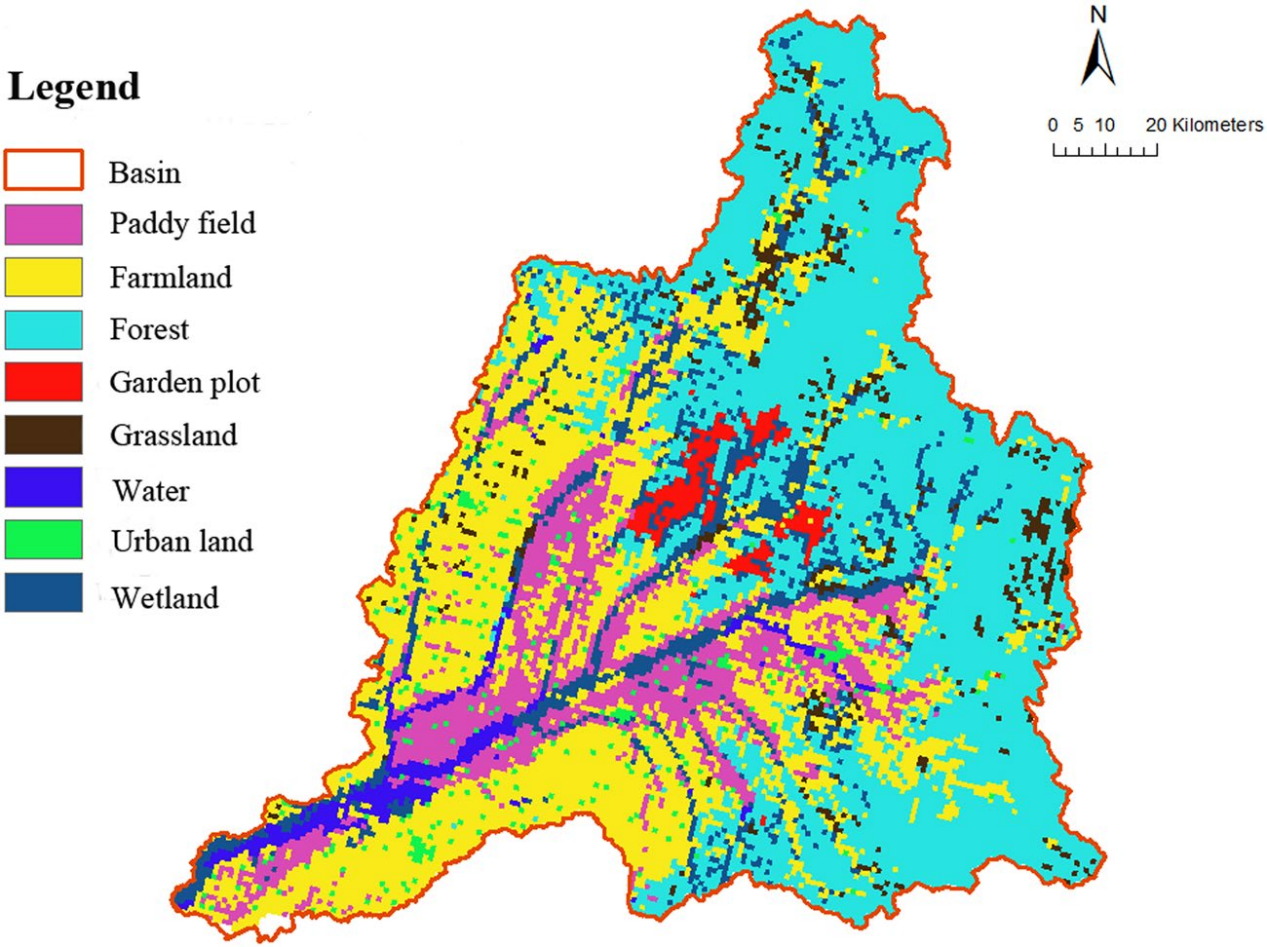
Land use distribution in the river basin.

According to 2015 land use remote sensing data (Figs. 11 and 12) combined with field survey results, the basin is mainly comprised of cultivated and forested land, accounting for more than 80% of the total land use. Forest land is concentrated in the upper reaches of the basin and the middle reaches are mainly cultivated. Following the confluence of tributaries in the lower reaches, residential land intensifies.

### SWAT model simulation

#### Model configuration and validation

Many input parameters are required for the SWAT model^34^, including digital elevation models (DEM), land-use area, soil type, meteorological data, and hydrological data^35^ as shown in Table 3.

**Table 3:** Data sources used in the Soil Water and Assessment Tool (SWAT) Model Simulation.

Data	Range Accuracy	Data Source
Digital elevation model	STRM 90 m	http://www.gscloud.cn/
Soil maps	1:1000000	Harmonized world soil database
Land use/cover	1:100000	http://www.resdc.cn/data.aspx?DATAID = 99
Weather data	CMADS (2008–2016)	http://westdc.westgis.ac.cn
Runoff	2008–2016	Hydrographic office

Due to the number of parameters in the SWAT model, individual calibration of parameters is difficult. Therefore, the sensitivity analysis method is generally used to determine the sensitivity of model parameters. Those parameters that have a large influence on model simulation results are selected using the SWAT-CUP sensitivity analysis tool to reduce the workload during model calibration and verification as shown in Table 4 ^36^.

**Table 4:** Results of SWAT-CUP Sensitivity analysis.

Rank	Runoff	Sediment	Total N	Total P
1	SMTMP.bsn	SPCON	NPERCO	PPERCO
2	SMFMX.bsn	CN2	SOL_ORGN	PHOSKD
3	TIMP.bsn	SPEXP	USLE_P	CN2
4	GW_DELAY.gw	SLOPE	SOL_NO3	SOL_ORGP
5	ALPHA_BF.gw	ULSE_P	CN2	SLOPE
6	ESCO.hru	SOL_Z	BIOMIX	USLE_P
7	ALPHA_BNK.rte	GWQMN	SLOPE	USLE_C

The hydrological cycle forms the basis of the hydrological model; however, rainfall and runoff are the driving forces of non-point source pollution. Therefore, the calibration and verification sequence of SWAT model parameters are runoff, sediment, and water quality. The model was calibrated spatially from the upper to lower reaches at Tieli, Sifang, and Qinjia stations.

The accuracy of the model simulation results can directly reflect the applicability of the model in a study area. Here, the relative error (PBIAS), the deterministic coefficient (R^2^), and the Nash efficiency coefficient (NSE) were used to evaluate model simulation results^37^.

5 $${\rm{NSE}}=1-[\frac{{\sum }_{i=1}^{n}{({Q}_{i}^{obs}-{Q}_{i}^{sim})}^{2}}{{\sum }_{i=1}^{n}{({Q}_{i}^{obs}-{Q}^{obs})}^{2}}]$$

6 $$PBIAS=[\frac{{\sum }_{i=1}^{n}({Q}_{i}^{obs}-{Q}_{i}^{sim})\ast 100}{{\sum }_{i=1}^{n}{Q}_{i}^{obs}}]$$

7 $${{\rm{R}}}^{2}={[\frac{{\sum }_{i=1}^{n}({Q}_{i}^{obs}-{Q}^{obs})\times ({Q}_{i}^{sim}-{Q}^{sim})}{\sqrt{{\sum }_{i=1}^{n}{({Q}_{i}^{obs}-{Q}^{obs})}^{2}}\sqrt{{\sum }_{i=1}^{n}{({Q}_{i}^{sim}-{Q}^{sim})}^{2}}}]}^{2}$$

Where ${Q}_{i}^{obs}$ is the runoff observation value, ${Q}_{i}^{sim}$ is the runoff simulation value, ${Q}^{obs}$ is the mean observed value, and ${Q}^{sim}$ is the mean simulated value. When the relative error between simulated and measured runoff is within ±20%, NSE > 0.5, and R^2^ > 0.6; and the relative error between simulation and measured sediment is within ±30%, NSE > 0.5, and R^2^ > 0.6, the SWAT model is considered consistent with observations and can be used for simulation of the basin^38,39^.

We can see from the Tables 5–7, simulation results for the upper reaches (Sifang and Tieli stations) are better as compared to those of Qinjia station (lower reaches) because runoff from the upper reaches is abundant, presenting a natural river form. However, downstream water conservancy facilities, including reservoirs and river dams mean that downstream water supply is insufficient, leading to intermittent flow in many places. Hence, upstream simulation results are more accurate results due to the significant influence of human activities downstream. Notwithstanding, model simulation results are in general accord with SWAT model requirements and can be applied to the Hulan River Basin.

**Table 5:** Simulation evaluation of monthly runoff.

Index	Tieli		Sifangtai		Qinjia	
	Calibration (2010–11)	Verification (2012)	Calibration (2010–11)	Verification (2012)	Calibration (2010–12)	Verification (2014)
NSE	0.741	0.769	0.753	0.643	0.721	0.713
R^2^	0.743	0.81	0.723	0.756	0.75	0.693
PBIAS	0.06	0.30	0.06	0.23	0.605	0.356

**Table 6:** Simulation evaluation of sediment transport.

Index	Tieli		Sifangtai		Qinjia	
	Calibration (2010–13)	Validation (2014)	Calibration (2010–13)	Verification (2014)	Calibration (2010–13)	Verification (2014)
NSE	0.661	0.739	0.673	0.643	0.641	0.613
R^2^	0.725	0.726	0.753	0.714	0.638	0.624
PBIAS	0.354	0.297	0.31	0.40	0.613	0.423

**Table 7:** Simulation evaluation of total phosphorus.

Index	Sifangtai		Qinjia	
	Calibration (2010–2011)	Verification (2012)	Calibration (2010–2012)	Verification (2014)
NSE	0.653	0.643	0.621	0.635
R^2^	0.611	0.616	0.672	0.641
PBIAS	0.41	0.43	0.417	0.55

#### SWAT model results

**Figure 13:**
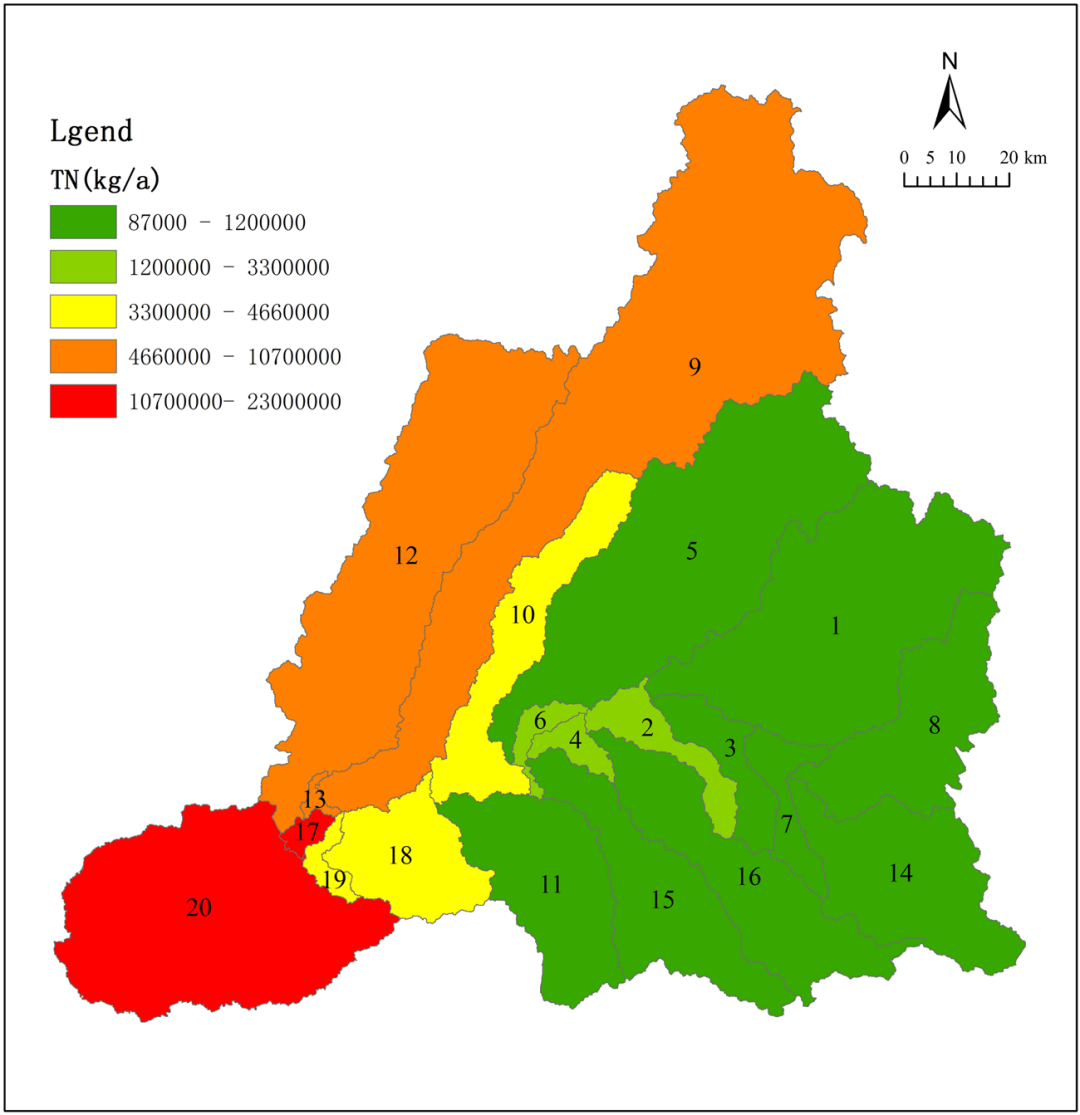
Simulated total nitrogen load flux.

**Figure 14:**
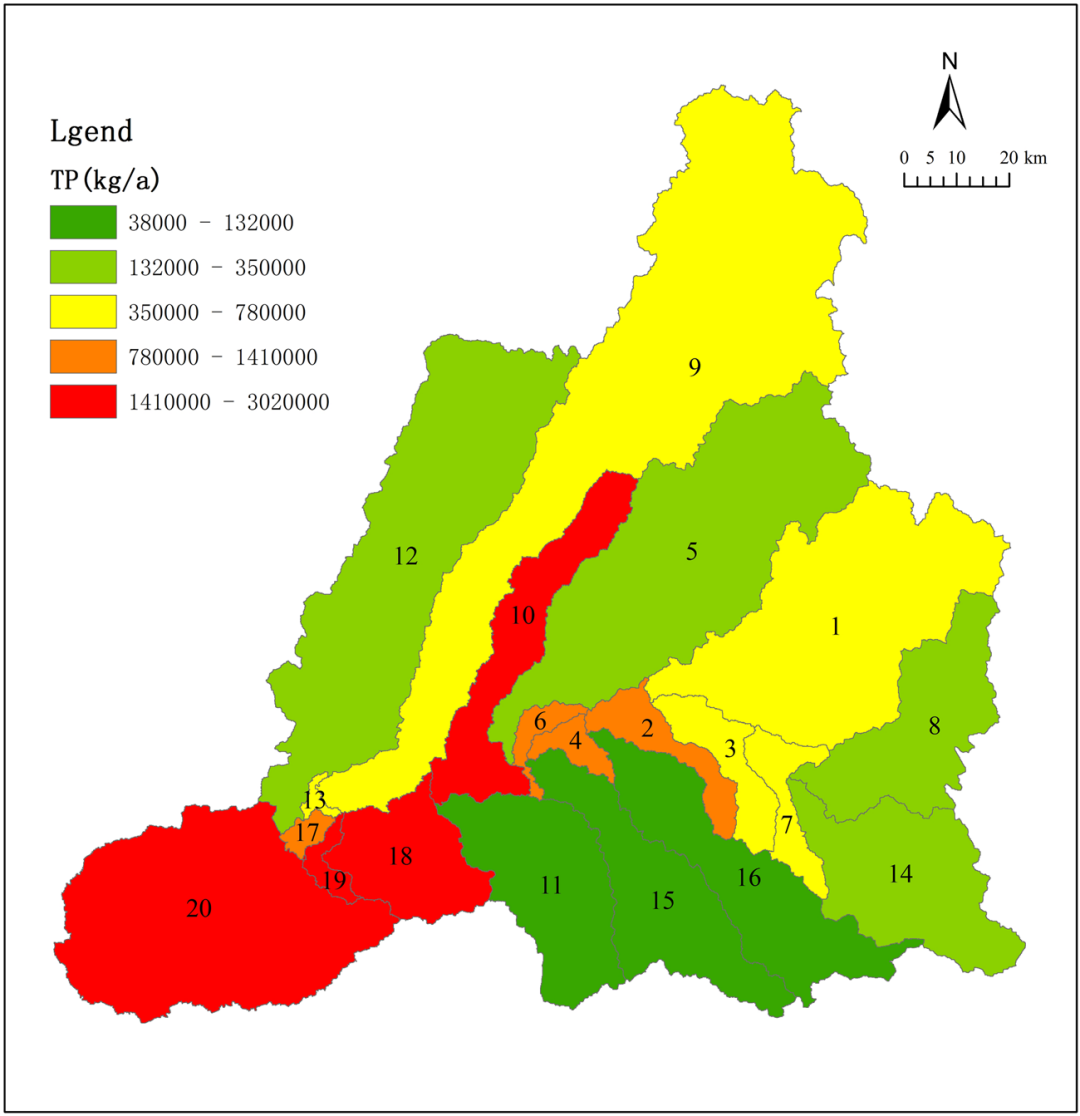
Simulated total phosphorus load flux.

Figures 13 and 14 show the simulated distribution of total N and P. It is apparent that SWAT model simulations of non-point source pollution loading in the upstream (downstream) is relatively low (high). In the Keyin and Numin River sub-basins, the non-point source pollution load of total N is relatively high. Conversely, the non-point source pollution load of total P is relatively high in the Ougen, Yijimi and Xiaohulan River sub-basins.

## Discussion

The variation of water chemistry from upstream to downstream (Fig. 2) shows that the dissolution of magnesium minerals gradually increases, while the dissolution of carbonate rocks gradually decreases, indicating a difference in lithology of the tributary source rock or riverbed sediments from upstream to downstream within the Hulan river basin. Due to the self-cleaning function of nitrogen in the water, there is no obvious accumulation of total N in the middle and lower reaches. Because of the poor self-purification function of P in water, total P gradually accumulates downstream, and the water quality deteriorates from Class III to Class V or worse (Fig. 8). In summary, the surface waters of the Hulan River Basin tend to be medium–weakly alkaline with a low degree of mineralization. HCO_3_-Ca and HCO_3_•Cl-Mg•Na (HCO_3_•Cl-Na•Ca) are the main chemical types, and the ion composition of each region changes regularly. In terms of drinking water safety, total N and P concentrations exceed the standard.

Stratigraphic rock (soil) minerals determine the source of groundwater chemical composition through water-rock interaction, which is the material basis of chemical components in surface water. The atmosphere is filled with CO_2_ of different origins, forming a gas-liquid-mineral three-phase system, and chemical reactions of atmospheric precipitation with certain chemical components and soil minerals occur at the contact surface of gaseous CO_2_ with water as follows:

8 $${{\rm{CO}}}_{2}({\rm{g}})\rightleftharpoons {{\rm{CO}}}_{2}({\rm{aq}})$$

9 $${{\rm{CO}}}_{2}({\rm{aq}})+{{\rm{H}}}_{2}{\rm{O}}\rightleftharpoons {{\rm{H}}}_{2}{{\rm{CO}}}_{3}$$

10 $${{\rm{H}}}_{2}{{\rm{CO}}}_{3}\rightleftharpoons {{\rm{HCO}}}_{3}^{-}+{{\rm{H}}}^{+}$$

11 $${{\rm{HCO}}}_{3}^{-}\rightleftharpoons {{\rm{CO}}}_{3}^{2-}+{{\rm{H}}}^{+}$$

12 $${{\rm{H}}}_{2}{\rm{O}}\rightleftharpoons {{\rm{OH}}}^{-}+{{\rm{H}}}^{+}$$

13 $${{\rm{OH}}}^{-}+{{\rm{CO}}}_{2}({\rm{aq}})\rightleftharpoons {{\rm{HCO}}}_{3}^{-}$$

According to qualitative lithology analysis, the main rock minerals are carbonates and silicates. On the basis of proton (H^+^) generation in the water and gas system, water-rock interaction occurs during phreatic water flow through the pores of the unconfined aquifer. The dissolution of carbonate and aluminosilicate minerals provides a source of Ca^2+^ and Mg^2+^ in the phreatic water, and the dissolution of rock salt provides a source of Na^+^, K^+^ and Cl^−^ as follows:

14 $${{\rm{CaCO}}}_{3}+{{\rm{H}}}^{+}\rightleftharpoons {{\rm{Ca}}}^{2+}+{{\rm{HCO}}}_{3}^{-}$$

15 $${\rm{CaMg}}{({{\rm{CO}}}_{3})}_{2}+2{{\rm{H}}}^{+}\rightleftharpoons {{\rm{Ca}}}^{2+}+{{\rm{Mg}}}^{2+}+{{\rm{2HCO}}}_{3}^{-}$$

16 $${\rm{Na}}({\rm{K}}){\rm{Cl}}\rightleftharpoons {{\rm{Na}}}^{+}({{\rm{K}}}^{+})+{{\rm{Cl}}}^{-}$$

17 $$2{\rm{Na}}({\rm{K}}){{\rm{AlSi}}}_{3}{{\rm{O}}}_{8}+2{{\rm{CO}}}_{2}+11{{\rm{H}}}_{2}{\rm{O}}\rightleftharpoons 4{{\rm{H}}}_{4}{{\rm{SiO}}}_{4}+2{{\rm{HCO}}}_{3}^{-}+2{{\rm{Na}}}^{+}({{\rm{K}}}^{+})+{{\rm{Al}}}_{2}{{\rm{Si}}}_{2}{({\rm{OH}})}_{4}$$

Topography controls the spatial distribution of individual ionic components. The water-rock reaction dominated by leaching occurs in the upstream where the hydraulic gradient is large. Under leaching processes, some HCO_3_^−^ type substances in soil enter the river and migrate as runoff to form HCO_3_-Ca type water with a low TDS. The particle size downstream, where the hydraulic gradient is slow, becomes finer, resulting in enhanced evaporation and concentration; hence TDS is gradually increased. As individual tributaries continue to flow into the mainstream, the concentration of mainstream components will change accordingly. For example, the Hulan River main water chemistry type is HCO_3_•Cl-Ca; however, following the merging of the Yijimi River in the middle reaches, the water is of the type SO_4_•HCO_3_•Cl-Ca.

Land use (Fig. 9) and water quality data indicate that water quality in densely populated regions is poor. In particular, the COD content is high at sampling points 26 and 27 on the Gemuke River, 21 and 22 on the Keyin River, and the lower reaches of the Hulan River. Human activities and daily life produce large volumes of sewage which discharges into the water body, leading to a deterioration in water quality. Land use and land cover changes result in temporal and spatial variability in water cycling, quantity, and quality. With the increase in human activity in the Hulan River basin, land cover within the basin has changed from natural vegetation to cultivated land, resulting in higher levels of N and P due to the large-scale use of fertilizer, herbicides, and pesticides. Different types of cultivated land lead to different degrees of N and P pollution. For example, the dry land in Group B is planted with soybean and corn, while Group A is mainly dominated by paddy fields. Consequently, the water quality of Group B is significantly worse than Group A.

Due to the development of tourism and the lack of oversight, the flow of the Yijimi River is severely restricted by fallen trees and prefabricated panels, resulting in the serious deterioration of water quality. Furthermore, a dam was built for the municipal landscape in the Gemuke River within Qing’an County, reducing river flow and causing serious eutrophication and poor water quality downstream.

At present, the evolution and cause analysis of surface water and groundwater quality within coastal areas and lakes, the hydrological charactersitics of which are different from rivers, is also a research hotspot^40–43^. If future research on water quality can proceed from the scale of the hydrological cycle, such as inland cycles and ocean cycles, more progressive and satisfactory results will be achieved.

## Conclusion

The TDS of Group B is higher as compared to the Hulan River mainstream, which in turn is higher as compared to Group A. The ion concentrations of the rivers in the basin are gradually enriched downstream. Surface waters of the Hulan River basin display relatively low TDS, and are generally medium-weakly alkaline fresh water. The water chemistry type is dominated by HCO_3_-Ca and HCO_3_•Cl-Mg•Na (HCO_3_•Cl-Na•Ca), and the ion composition of each region changes regularly. In terms of drinking water quality, total N and P exceed water safety standards.

The water chemistry of the basin is mainly controlled by rock weathering. Water ions are mainly composed of silicate mineral weathering products, followed by carbonate mineral weathering products which corresponds well with the geology of the region. The upstream hydraulic gradient is large, and water-rock processes are dominated by leaching. Downstream, particles become finer and TDS is gradually increased under enhanced evaporation conditions and the decreased hydraulic gradient.

Increased human activity in the river basin has altered land cover from natural vegetation to cultivated land, resulting in water quality degradation. The content of N and P is generally high due to the large-scale use of fertilizer, herbicides, and pesticides. The degree of N and P pollution differs according to the type of cultivated land. The non-point source pollution load is relatively low upstream and increases downstream. In the Keyin and Numin River sub-basin, the non-point source pollution load of total N is relatively high. Conversely, in the Ougen, Yijimi, and Xiaohulan River sub-basin, the non-point source pollution load of total P is relatively high.

## Data Availability

The datasets generated and analysed during the current study are available from the corresponding author on reasonable request.
